# Flexible intramedullary nails with traction versus plaster cast for treating femoral shaft fractures in children: comparative retrospective study

**DOI:** 10.1590/S1516-31802013000100002

**Published:** 2013-02-01

**Authors:** Fabiano Prata do Nascimento, Cláudio Santili, Miguel Akkari, Gilberto Waisberg, Susana dos Reis Braga, Patrícia Maria Moraes de Barros Fucs

**Affiliations:** I MD, PhD. Head of the Pediatric Orthopedic Staff in the Itaim and Anália Franco Units of Hospital São Luiz, São Paulo, and Coordinator of the Orthopedic Staff in Hospital Estadual de Santo André, Faculdade de Medicina do ABC, Santo André, São Paulo, Brazil.; II MD, PhD. Adjunct Professor, Faculdade de Ciências Médicas, Irmandade da Santa Casa de Misericórdia de São Paulo, and Attending Physician, Pediatric Orthopedic Group, Santa Casa de Misericórdia de São Paulo, São Paulo, Brazil.; III MD, PhD. Head of the Pediatric Orthopedic Group in Santa Casa de Misericórdia de São Paulo, São Paulo, Brazil.; IV MD. Attending Physician in the Pediatric Orthopedic Group, Santa Casa de Misericórdia de São Paulo, São Paulo, Brazil.; V MD, MSc. Attending Physician in the Pediatric Orthopedic Group, Santa Casa de Misericórdia de São Paulo, São Paulo, Brazil.; VI MD, PhD. Adjunct Professor, Faculdade de Ciências Médicas, Irmandade da Santa Casa de Misericórdia de São Paulo, and Head of the Neuromuscular Disorders Group, Santa Casa de Misericórdia de São Paulo, São Paulo, Brazil.

**Keywords:** Femur, Femoral fractures, Bone nails, Child, Orthotic devices, Fêmur, Fraturas do fêmur, Pinos ortopédicos, Criança, Aparelhos ortopédicos

## Abstract

**CONTEXT AND OBJECTIVE:**

Femoral fractures are common in children between 2 and 12 years of age, and 75% of the lesions affect the femoral shaft. Traction followed by a plaster cast is universally accepted as conservative treatment. However, in some situations, a surgical approach is recommended. The objective here was to compare treatments for femoral shaft fractures using intramedullary nails (titanium elastic nails, TEN) versus traction and plaster casts in children. The hypothesis was that TEN might provide better treatment, with good clinical results in comparison with plaster casts.

**DESIGN AND SETTING:**

This retrospective comparative study was conducted in a public university hospital.

**METHODS:**

Sixty children with femoral fractures were evaluated; 30 of them underwent surgical treatment with TEN and 30 were treated conservatively using plaster casts. The patients’ ages ranged from 5 to 13 years (mean of 9 years).

**RESULTS:**

The mean duration of hospitalization was nine days for the surgical group and 20 days for the conservative group. The incidence of overgrowth in the patients treated with TEN was 60.0% and, for those treated conservatively, 13.3%. Partial weight-bearing was allowed after 3.5 weeks in the surgical group and after 9.6 weeks in the conservative group. New hospitalization was required for 90.0% in the surgical group and 16.7% in the conservative group. Patients treated with plaster casts presented higher incidence of complications, such as loss of reduction.

**CONCLUSIONS:**

The surgical method presented better results for children.

## RESUMO

**CONTEXTO E OBJETIVO:**

Fraturas femorais são comuns em crianças entre 2 e 12 anos de idade, e 75% das lesões acometem a diáfise. Tração seguida de aparelho gessado (“gesso”) é universalmente aceita como tratamento conservador. Entretanto, em algumas situações o tratamento cirúrgico é recomendado. O objetivo foi comparar o tratamento de fraturas diafisárias do fêmur com hastes intramedulares (*titanium elastic nails,* TEN) com tração e gesso em crianças. A hipótese era de que TEN pode ser melhor tratamento, com bons resultados clínicos em comparação com o gesso.

**TIPO DE ESTUDO E LOCAL:**

Este estudo retrospectivo e comparativo foi conduzido num hospital público universitário.

**MÉTODOS:**

Sessenta crianças com fraturas de fêmur foram avaliadas, 30 delas foram submetidas a tratamento cirúrgico com TEN e 30 foram tratadas de forma conservadora usando gesso. A idade dos pacientes variou de 5 a 13 anos (média de 9 anos).

**RESULTADOS:**

O tempo médio de internação foi de 9 dias para o grupo cirúrgico e 20 dias para o grupo conservador. A incidência de crescimento excessivo nos pacientes tratados com TEN foi de 60,0% e, para aqueles tratados de forma conservadora, 13,3%. Sustentação parcial de peso foi permitida após 3,5 semanas no grupo cirúrgico e após 9,6 semanas no grupo conservador. Houve 90,0% de novas internações no grupo cirúrgico e 16,7% no grupo conservador. Pacientes tratados com gesso apresentaram maior incidência de complicações, tais como perda de redução.

**CONCLUSÃO:**

O método cirúrgico apresentou melhores resultados para as crianças.

## INTRODUCTION

Femoral fractures are most common in children around the age of two years and around 12 years old, therefore with two peaks of incidence, and 75% of the lesions affect the femoral shaft.^1^ Skin or skeletal traction followed by a plaster cast is universally accepted as a conservative treatment.

However, in open and multiple fractures in children over 10 years of age, a surgical approach has been recommended. Intramedullary rods (rigid or semi-rigid),^2^^,^^3^ elastic stable intramedullary nails (ESIN) or simply titanium elastic nails (TEN) have been used very successfully in children under the age of 12 years. Intramedullary nails are better suited for transverse and/or short oblique fractures than for long oblique or comminuted fractures, which respond better to external fixation or to traction followed by a cast.^1^

We previously reported our first results using TEN in cases of femoral fractures in children from 5 to 14 years of age.^4^ This study compares those results with conservative treatment, in children of the same ages, especially focusing on the duration of hospitalization, discrepancies and deformities and the time taken to achieve weight bearing and the return to daily activities. The setting was a public university hospital in Brazil, where cost of treatment can be an issue and surgical treatment was only incorporated in the routine service ten years ago. The conservative method was represented by traction followed by a cast, and the surgical method by intramedullary fixation with TEN. The hypothesis was that intramedullary nails would offer an earlier return to daily activities with fewer complications compared with plaster casts.

## OBJECTIVE

To compare treatments for femoral shaft fractures using TEN, *versus* traction and plaster casts in children. The hypothesis was that TEN might provide better treatment, with good clinical results in comparison with plaster casts.

## METHODS

### Setting and patients

This retrospective comparative study was conducted in a public university hospital, covering the period between January 1995 and February 2004. All children between the ages of 5 and 14 years who were treated during this period for femoral shaft fractures using TEN or traction and cast, and with at least 24 months of follow-up, were included.

Before 2000, TEN was not used in Brazil and it was thus not available in public services: femoral shaft fractures were therefore treated conservatively. With the advent of TEN, it became possible to undertake a retrospective analysis comparing those previously treated with casts with those treated with nails. A total of 60 children (a convenience sample) with unilateral fracture of the femoral shaft during this period were evaluated. During the first part of the study period, all children underwent skin or skeletal traction before treatment. When nails became available in our public service, most children underwent surgery.

In this manner, 30 patients treated using TEN and 30 patients treated using traction followed by a cast during the study period were selected. Patients who presented diseases that could affect the normal anatomical and physiological characteristics of the skeleton, such as osteometabolic diseases, bone dysplasia or pathological fractures associated with neuromuscular syndromes or alterations were excluded. The institution’s Ethics Committee approved the study.

In 60.0% of the cases, the fracture was caused by pedestrian-automobile collisions; 21.6% were due to falls from heights; 10% were the result of motor-vehicle accidents; in 5%, objects fell onto the thigh; 1.7% of the patients suffered bicycle accidents; and 1.7% were child abuse victims. Associated lesions were found in 22% of the patients with femoral fractures. Of these, 30.7% had tibial fractures.

Regarding the patients treated with TEN, the fracture was transverse in 60.0% of the cases and oblique in 26.7%. In the conservative group, 36.7% of the fractures were transverse and 40.0% were oblique (P = 0.264). The transverse and oblique fractures represented 81.7% of all the fractures. There were only two open fractures (grade II of Gustilo and Anderson), in the surgical group. Treatments casts were placed at least five days after fracture occurrence (mean: 18.7 days).

**Figure 1.**
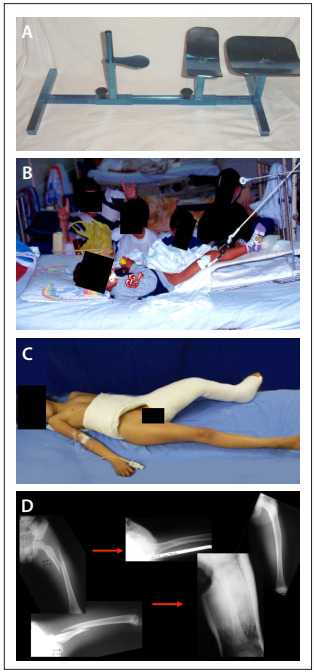
Children’s orthopedic table (A) for traction (B) and cast (C), for children with femoral shaft fractures; and radiographs showing the reduction control before and after the cast was applied (D).

The cast for the conservative treatment was constructed on a children’s orthopedic table, with the hips flexed at between 30 and 45 degrees, with semi-flexion of the knee and inclusion of the foot. Following this, radiographs were taken for reduction control. The cast was then made after evaluating the adjacent soft tissues and the radiographic results (i.e. to investigate shortening or formation of bone callus) (Figure 1).

**Figure 2.**
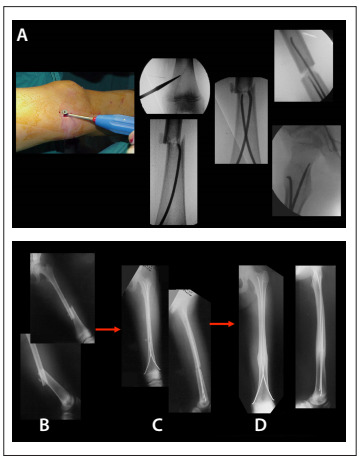
Fixation of flexible intramedullary titanium elastic nails (TEN) in a child with femoral shaft fracture: procedure on a radiotransparent table, with the patient in supine position (A) and radiographs taken initially (B), in the immediate postoperative period (C) and finally (D).

The patients treated with TEN (made by Synthes, known as “Nancy nails”) were referred to the surgical center and operated on as soon as their clinical conditions were favorable. The mean delay between injury and surgical fixation was 5.3 days. Associated injuries lengthened this period; for instance, one patient with fractures of the clavicle and ipsilateral tibia was operated after 14 days; all the others were treated within 10 days at most. The patients were placed on a radiotransparent table in the supine position. The diameter of each nail was roughly 40% of the respective medullary canal diameter (at the isthmus). The nails were introduced with the aid of an image intensifier, in a retrograde fashion, two to three centimeters proximally from the femoral distal growth cartilage (Figure 2). No casts were used for complementary immobilization. Depending on the fracture characteristics and its reduction, early weight-bearing and joint movement were allowed, especially of the knee, and the patients were encouraged to do so from the first postoperative day onwards.

In the subsequent evaluations, in addition to physical examination, radiographs of the affected limb in the anterior-posterior and lateral views were produced, and scanometry of the lower limbs was performed. Angular deformities (in degrees), discrepancies (in centimeters), initial and final shortening and excessive growth were measured. Other variables were also analyzed, such as the duration of traction and hospitalization, time taken for the patient to be able to bear weight on the limb and return to his daily activities, time taken for consolidation to be achieved, presence of acute and late complications, number of subsequent hospitalizations, complaints and length of follow-up. Between six and eight months after fracture healing and remodeling, the nails were removed surgically, in a single-day hospitalization period, and the children could walk freely thereafter. Children treated using casts had the cast removed after consolidation, which took approximately two months to achieve. They were then allowed to bear weight partially two weeks later. Use of crutches was ended after fracture healing.

### Data analysis

The data were analyzed as absolute and relative frequencies for quantitative measurements, and were presented as means, frequencies (%) and standard deviations. Comparisons were made using the Fisher, Mann-Whitney tests, Barlett and Pearson chi-square test. Analysis of variance (ANOVA) was also used.

## RESULTS

### Patients’ ages

The patients’ ages ranged from 5.0 to 13.5 years (mean: 9 years). The patients in the surgical group had a mean age of 9.6 years and the patients in the conservative group had a mean age of 8 years (analysis of variance, ANOVA; P = 0.004). There were 41 male patients (68.4%). In the surgical group, the gender distribution was even, but in the conservative group, there was a preponderance of male patients (83.3%).

### Lengths of hospitalization and follow-up and time taken to return to activities

**Graph 1.**
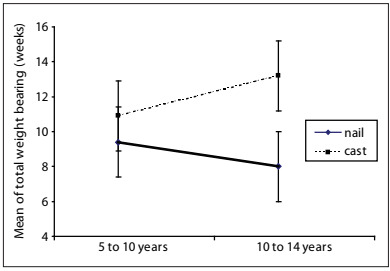
Mean and standard error of load (in weeks), according to the treatment (surgical or with cast), for femoral shaft fractures in children in two age groups.

The minimum length of follow-up was 24 months for the surgical patients (mean: 35.4 months) and 59.0 months for those treated using casts. The mean duration of hospitalization was significantly different between the groups: 9.4 days for the surgically treated children and 20.5 days for the conservatively treated children (ANOVA; P < 0.001). The times taken for healing were also significantly different: 7.7 weeks for consolidation in the surgical group and 9.3 weeks in the conservative group (ANOVA; P = 0.005). The mean duration of traction was 5.3 days for the surgical group, with a maximum of 14 days and a mean of 18.4 days for the conservative group, with a maximum of 40 days. The mean time taken for the patients to return to their activities was 3.7 weeks (ranging from one week to ten weeks) for the surgical group and 9.5 weeks (ranging from six weeks to 16 weeks) for the conservative group (P < 0.001). Partial loading was allowed after 3.5 weeks, on average, for the surgical group and after 9.6 weeks for the conservative group (Mann-Whitney; P < 0.001). There was a relationship between increasing age and longer time taken for weight-bearing on the fractured limb to be allowed among the patients who were treated conservatively (i.e. with casts). On the other hand, for those treated with TEN, the time taken for this remained relatively constant: for younger patients (5 to 9 years of age), the average was 3.4 weeks, and for those aged 10 to 14 years, 3.8 weeks (ANOVA; P = 0.000). The average time taken for total weight-bearing to be allowed was 8.8 weeks for the patients treated with nails and 11.3 weeks for the patients treated with casts (ANOVA; P = 0.007). For the conservative method, the total time taken for weight-bearing to be allowed increased with increasing age; for the surgical method, the opposite was seen (Graph 1). The percentage of patients hospitalized was 90.0% in the surgical group and 16.7% in the conservative group. None of the patients suffered a repeated fracture in either group. Most hospitalizations after surgical treatment were preplanned, in order to remove the nail.

In the surgical group, one patient presented migration of the nail (the tip of the nail was cut off in the new surgery); another patient fell, thereby losing his reduction (new surgery was indicated); a third patient suffered soft-tissue irritation (the nail had to be removed early on). Among these three, only the case of migration required new hospitalization that had not been preplanned. The patient who fell was a girl who tried to walk during the first hospitalization and fell from her bed. In the conservative group, three patients lost their reduction: one at six weeks; one who started walking before consolidation had been achieved; and one case in which the patient removed the pelvic portion of the cast. These events required a second hospital admission and, in the case of the surgically treated patients, a third admission. A significant difference was found between the groups regarding the total number of hospitalizations, including those for nail removal (Pearson’s chi-square test; P < 0.001). All the surgical patients underwent closed reduction. This was possible because of the traction that was firstly performed on all the patients (28 patients underwent skeletal traction and two patients underwent cutaneous traction).

### Shortening, overgrowth and deformities

The mean initial shortening, prior to treatment, was 2 cm, ranging from 0.5 to 4.5 cm for the entire group, from 0.5 to 3 cm in the conservative group and from 0.5 to 4.5 cm in the surgical group. Shortening, after a period of at least 24 months, occurred in 6.7% of the patients in the surgical group and in 63.3% of the patients in the conservative group (Pearson’s chi-square test; P < 0.001). In the surgical group, the mean shortening was 0.25 cm and in the conservative group the mean was 1.14 cm (ANOVA; P = 0.133), with no significant difference between the means of the two groups. There was overgrowth in 60.0% of the patients in the surgical group and 13.3% in the conservative group (Pearson’s chi-square test; P < 0.001). The mean overgrowth was not statistically different (ANOVA; P = 0.072) and was 0.66 cm (range: 0.25 to 1.50 cm) in the surgical group and 1.06 (range: 0.05 to 1.50 cm) in the conservative group.

Every patient presented some type of deformity. The average posterior angulation and varus and valgus deformities did not exceed 10 degrees. The mean and standard deviation of the anterior angulation deformity was 6.5 degrees for patients treated surgically and 12.1 degrees for the patients treated using traction and cast. Complaints about the treatment were registered in the medical records, from 10% of the patients treated with nails and from 16.6% of the patients treated with casts.

Table 1 summarizes the results.

**Table 1.** Demographic and clinical data on the 60 children treated for femoral shaft fractures with cast or TEN (titanium elastic nails)

	Traction + cast	TEN	P
Age	8.0 years	9.6 years	P = 0.004
Duration of traction	18.7 days	5.3 days	P < 0.001
Duration of hospitalization	20.5 days	9.4 days	P < 0.001
Time taken to heal	9.3 weeks	7.7 weeks	P = 0.005
Shortening: rate of occurrence	63.3%	6.7%	P < 0.001
Shortening: mean	1.14 cm	0.25 cm	P = 0133
Overgrowth: rate of occurrence	13.3%	60%	P < 0.001
Overgrowth: mean	1.06 cm	0.66 cm	P = 0.072
Varus	5.9 degrees	4.0 degrees	P = 0.976
Valgus	10.0 degrees	6.7 degrees	P = 0.094
Anterior angulation	12.1 degrees	6.5 degrees	P < 0.001
Posterior angulation	5.3 degrees	2.3 degrees	P = 0.172
Partial weight-bearing	9.6 degrees	3.5 degrees	P < 0.001
Full weight-bearing	11.3 degrees	8.8 degrees	P = 0.007
Return to daily activities	9.5 weeks	3.7 weeks	P < 0.001

## DISCUSSION

### Age and indication for treatments

Conservative treatment using an early cast is indicated for children with femoral fractures under the age of 11 years,^5^^,^^6^^,^^7^^,^^8^^,^^9^^,^^10^ but some authors advocate this treatment method only for children under six years of age.^11^^,^^12^ Traction may be used before applying the cast, in view of the possibility of significant limb shortening and bad alignment in children over the age of five or six years.^13^ Among the disadvantages from conservative methods are limb discrepancies or angle deviations, compartment syndrome due to skin traction and possible psychological harm. Moreover, longer hospitalization periods and consequent higher costs are attributed to conservative treatment.^2^^,^^14^^,^^15^ The advantages of such treatment are that it is available in all healthcare services and that it eliminates the risks that are inherent to surgery.

Surgical treatment in our service was only recently introduced into routine practice, such that observation of the dates of treatment (conservative cases from 1995 to 2004 and surgical cases from 2000 to 2004) in our sample allow us to say that there has been a gradual and natural transition towards choosing surgery. Today, surgery is the first choice in our pediatric service and this study provides an evaluation of the benefits that this change of approach towards femoral shaft fractures has brought.

Treatments using nails for fixation have been indicated for patients between the ages of 4 and 17 years, although Bopst et al.^16^ more recently reported an indication for children as young as 1.5 years of age and Simanovsky et al.^17^ for those aged three years and over. This age group includes the phase at which these patients go to school, and thus, independence during treatment is important for these patients. By reducing hospitalization time, children may return to school earlier, thereby avoiding social isolation and the need for extra care, such as the care needed to maintain hygiene when individuals are treated using casts.^4^^,^^18^^,^^19^^,^^20^^,^^21^ TEN or semi-rigid Ender rods for use in cases of transverse and short oblique fractures has been advocated as the best approach for children over the age of five years. These nails make it possible to control limb length and avoid growth cartilage lesions, and they also shorten the duration of hospitalization and allow faster recovery.^10^^,^^18^^,^^19^^,^^20^^,^^21^^,^^22^

In our study, TEN was mainly indicated for transverse and short oblique fractures, and in patients over the age of five years. Some patients had comminuted and spiral fractures and, in order to control the alignment of one of the comminuted fractures, three nails had to be applied.

The length of time under traction is directly associated with the duration of hospitalization, in cases in which a femoral fracture is the most significant lesion. Newton and Mubarak^23^ reported that their minimum hospitalization time prior to cast placement was 20.6 days for skin traction, 20.8 days for skeletal traction, 8.5 days for intramedullary nails and 2.5 days for early cast placement. Ligier et al.^20^ and Heinrich et al.^18^ reached different results: they analyzed fractures treated using flexible rods and found that the duration of hospitalization ranged from 4.5 to 8 days. Our patients treated with traction and cast presented significantly longer mean hospitalization times (20.5 days *versus* 9.4 days). One of the reasons for this may have been that, in our hospital, at least five days pass between fracture occurrence and cast placement.

It also should be taken into consideration that all cases treated surgically required another period of hospitalization in order to remove the nails. We chose to remove the nails six to eight months after their implantation, which is in accordance with reports from other authors, such as Flynn et al.^19^ and Buford et al.^24^ At this time, the fracture presents very solid union. Ligier et al.^20^ recommends nail removal three months after surgery.

In addition to the hospitalization for nail removal, we had one case that needed yet another hospitalization in order to cut the tip off one nail that had migrated. In the conservative group, additional hospitalizations were needed because of loss of positioning and reduction, as well as one for cast removal. A significant difference between the groups was found regarding the number of hospitalizations: some of the hospital admissions were not expected, but the majority were due to nail removal. An analysis by Santili et al.^25^ that included pediatric patients within our setting showed that conservative treatment of femoral shaft fractures was 22.5% more expensive than surgical treatment with flexible nails. We did not perform a cost analysis in this study, but the incidence of infections and other complications, which could have an impact on cost, was zero in our sample of surgical patients.

With regard to consolidation and return to activities, Ligier et al.^20^ and Saseendar et al.^26^ reported that elastic movement promoted faster and more abundant bone callus formation. Stans et al.^27^ reported that consolidation was faster using flexible rods than using external fixation. In our patients, the mean time taken for fracture consolidation to be achieved was 1.6 weeks shorter in the surgical cases than in the conservatively treated cases. Some authors (Stans et al.,^27^ Reeves et al.^28^ and Staheli and Sheridan^8^) considered that the fracture had healed and then removed the cast after eight weeks, on average, while others^11^ reported removing it after six weeks. The time taken to return to school among the patients treated with TEN, walking with the aid of crutches (i.e. partial loading), has ranged from two days to four weeks, and for full loading, from three to 11 weeks.^4^^,^^14^^,^^15^^,^^20^^,^^21^^,^^26^^,^^29^ Concerning weight-bearing and the return to normal activities in the present study, the surgical method allowed partial loading before consolidation and was earlier than with conservative treatment. This difference was highly significant (ANOVA; P < 0.001). Partial and full weight-bearing were allowed, on average, after 3.5 and 8.8 weeks for the surgical group, and after 9.6 and 11.3 weeks for the conservative group, respectively.

Therefore, there was a difference in partial load-bearing between the two methods, of approximately six weeks (ANOVA; P < 0.001). Concerning age, the time taken for partial and total loading to be allowed increased with increasing age among the patients treated with casts, while there was a reduction in the time taken for total loading to be allowed among those treated with TEN. The explanation for this is that the older patients probably assimilated the orientations for crutch use better. The definition for the return to daily activities was taken to be when the patient was able to walk on his own, with crutches, which exactly corresponded to when partial weight-bearing was allowed.

### Shortening, overgrowth and deformities

A consensus exists in the literature that the final shortening of the limb is produced by the initial shortening combined with the patient’s potential for growth, which is greater in younger children. Cadman and Neer^29^ considered that a maximum of 3 cm of shortening was acceptable, Czertak and Hennrikus^11^ considered that up to 2.5 cm was acceptable and Staheli^30^ and Buehler et al.^5^ considered that up to 1.5 cm was acceptable. In our group, the method using traction and cast caused greater shortening (mean of 1.14 cm), occurring in 63.3% of the patients in this group, in comparison with the surgical group, in which this occurred in only 6.7% of the patients (mean of 0.25; Pearson’s chi-square test, P < 0.001). Despite the differences between the groups, these values were clinically very well tolerated (ANOVA = 0.133). Only one patient had a final shortening of 4.0 cm. As observed in the literature,^18^^,^^20^ significantly greater overgrowth also occurred in our patients treated with TEN. This was present in 60.0% of the cases, whereas it was only present in 13.3% of the cases in the conservative group.

However, even though the frequency of overgrowth was different between the groups, there was no statistically significant difference in the amount of overgrowth between the two methods used (P = 0.072), with a mean of 0.66 cm for the surgical group and 1.06 cm for the conservative group. These results are compatible with what has been reported in the literature.^13^^,^^18^^,^^19^^,^^20^^,^^29^^,^^31^^,^^32^

All the patients in the present study presented some type of deformity. The means for the varus, valgus, posterior and rotational deviations were less than ten degrees with both methods. According to Flynn et al.,^19^ angles of less than ten degrees are considered satisfactory; therefore, we can consider that the results from our patients are in conformity with the literature standards. We had high incidence of anterior angulation, with means of 6.5 degrees for the surgical group and 12.1 degrees for the conservative group. It must be emphasized that in the sagittal plane, the measurement included the physiological angulation of the femur. Some authors have reported losses of reduction and angular deviation when treating children with more than 45 kg of body weight. Care is required when indicating flexible intramedullary nails for patients who are obese and closer to skeletal maturity, and an indication for an interlocking pediatric nail with a lateral entry point should be considered.^5^^,^^33^^,^^34^^,^^35^ In order to provide better control over the alignment of unstable fractures, such as comminuted, long oblique or spiral fractures, and in cases of fractures in heavy patients, a new device (end caps) was designed by the AO expert group to address these complications.^36^

This study had a retrospective design, and used a convenience sample, which were limitations of the study. The different characteristics of the two groups of patients were also a limitation. Nonetheless, this study points towards the important and already-suspected hypothesis that femoral shaft fractures in children can be better treated with surgery. This is a proper scenario within which a randomized controlled trial could be developed in order to obtain reliable answers, without bias.

## CONCLUSION

Patients older than five and younger than 14 years of age, with femoral shaft fractures treated using a flexible intramedullary method, returned to daily activities and to school earlier, with shorter periods of traction and hospitalization and less limb shortening and a lower rate of loss of reduction, compared with those treated with casts. Both methods showed few complications or problems relating to alignment.

## References

[1] Flynn JM, Schwend RM (2004). Management of pediatric femoral shaft fractures. J Am Acad Orthop Surg.

[2] Kirby RM, Winquist RA, Hansen ST (1981). Femoral shaft fractures in adolescents: a comparison between traction plus cast treatment and closed intramedullary nailing. J Pediatr Orthop.

[3] Mann DC, Weddington J, Davenport K (1986). Closed Ender nailing of femoral shaft fractures in adolescents. J Pediatr Orthop.

[4] Nascimento FP, Santili C, Akkari M (2010). Short hospitalization period with elastic stable intramedullary nails in the treatment of femoral shaft fractures in school children. J Child Orthop.

[5] Buehler KC, Thompson JD, Sponseller PD (1995). A prospective study of early spica casting outcomes in the treatment of femoral shaft fractures in children. J Pediatr Orthop.

[6] Irani RN, Nicholson JT, Chung SM (1976). Long-term results in the treatment of femoral-shaft fractures in young children by immediate spica immobilization. J Bone Joint Surg Am.

[7] Martinez AG, Carroll NC, Sarwark JF (1991). Femoral shaft fractures in children treated with early spica cast. J Pediatr Orthop.

[8] Staheli LT, Sheridan GW (1977). Early spica cast management of femoral shaft fractures in young children. A technique utilizing bilateral fixed skin traction. Clin Orthop Relat Res.

[9] Sugi M, Cole WG (1987). Early plaster treatment for fractures of the femoral shaft in childhood. J Bone Joint Surg Br.

[10] Templeton PA, Wright JG (1998). Femoral shaft fractures: North American and European perspectives. Current Orthopaedics.

[11] Czertak DJ, Hennrikus WL (1999). The treatment of pediatric femur fractures with early 90-90 spica casting. J Pediatr Orthop.

[12] Illgen 2nd R, Rodgers WB, Hresko MT (1998). Femur fractures in children: treatment with early sitting spica casting. J Pediatr Orthop.

[13] Viljanto J, Linna MI, Kiviluoto H, Paananen M (1975). Indications and results of operative treatment of femoral shaft fractures in children. Acta Chir Scand.

[14] Kissel EU, Miller ME (1989). Closed Ender nailing of femur fractures in older children. J Trauma.

[15] Timmerman LA, Rab GT (1993). Intramedullary nailing of femoral shaft fractures in adolescents. J Orthop Trauma.

[16] Bopst L, Reinberg O, Lutz N (2007). Femur fracture in preschool children: experience with flexible intramedullary nailing in 72 children. J Pediatr Orthop.

[17] Simanovsky N, Porat S, Simanovsky N, Eylon S (2006). Close reduction and intramedullary flexible titanium nails fixation of femoral shaft fractures in children under 5 years of age. J Pediatr Orthop B.

[18] Heinrich SD, Drvaric DM, Darr K, MacEwen GD (1994). The operative stabilization of pediatric diaphyseal femur fractures with flexible intramedullary nails: a prospective analysis. J Pediatr Orthop.

[19] Flynn JM, Hresko T, Reynolds RA (2001). Titanium elastic nails for pediatric femur fractures: a multicenter study of early results with analysis of complications. J Pediatr Orthop.

[20] Ligier JN, Metaizeau JP, Prévot J, Lascombes P (1988). Elastic stable intramedullary nailing of femoral shaft fractures in children. J Bone Joint Surg Br.

[21] Vrsansky P, Bourdelat D, Al Faour A (2000). Flexible stable intramedullary pinning technique in the treatment of pediatric fractures. J Pediatr Orthop.

[22] Huber RI, Keller HW, Huber PM, Rehm KE (1996). Flexible intramedullary nailing as fracture treatment in children. J Pediatr Orthop.

[23] Newton PO, Mubarak SJ (1994). Financial aspects of femoral shaft fracture treatment in children and adolescents. J Pediatr Orthop.

[24] Buford D, Christensen K, Weatherall P (1998). Intramedullary nailing of femoral fractures in adolescents. Clin Orthop Relat Res.

[25] Santili C, Akkari M, Waisberg G (2005). Tratamento incruento das fraturas diafisárias do fêmur nas crianças [Bloodless treatment of femoral diaphyseal fractures in children]. Acta Ortop Bras.

[26] Saseendar S, Menon J, Patro DK (2010). Treatment of femoral fractures in children: is titanium elastic nailing an improvement over hip spica casting?. J Child Orthop.

[27] Stans AA, Morrissy RT, Renwick SE (1999). Femoral shaft fracture treatment in patients age 6 to 16 years. J Pediatr Orthop.

[28] Reeves RB, Ballard RI, Hughes JL (1990). Internal fixation versus traction and casting of adolescent femoral shaft fractures. J Pediatr Orthop.

[29] Cadman EF, Neer CS (1957). Treatment of fractures of the femoral shaft in children. J Am Med Assoc.

[30] Staheli LT (1967). Femoral and tibial growth following femoral shaft fracture in childhood. Clin Orthop Relat Res.

[31] Aronson J, Tursky EA (1992). External fixation of femur fractures in children. J Pediatr Orthop.

[32] Kregor PJ, Song KM, Routt ML (1993). Plate fixation of femoral shaft fractures in multiply injured children. J Bone Joint Surg Am.

[33] Hunter JB (2005). Femoral shaft fractures in children. Injury.

[34] Keeler KA, Dart B, Luhmann SJ (2009). Antegrade intramedullary nailing of pediatric femoral fractures using an interlocking pediatric femoral nail and a lateral trochanteric entry point. J Pediatr Orthop.

[35] Li Y, Stabile KJ, Shilt JS (2008). Biomechanical analysis of titanium elastic nail fixation in a pediatric femur fracture model. J Pediatr Orthop.

[36] Nectoux E, Giacomelli MC, Karger C, Gicquel P, Clavert JM (2008). Use of end caps in elastic stable intramedullary nailing of femoral and tibial unstable fractures in children: preliminary results in 11 fractures. J Child Orthop.

